# Heuristic Methods for Finding Pathogenic Variants in Gene Coding Sequences

**DOI:** 10.1161/JAHA.112.002642

**Published:** 2012-10-25

**Authors:** Monique Ohanian, Robyn Otway, Diane Fatkin

**Affiliations:** Molecular Cardiology Division, Victor Chang Cardiac Research Institute, Sydney, New South Wales, Australia (M.O., R.O., D.F.); Cardiology Department, St Vincent's Hospital, Sydney, New South Wales, Australia (D.F.); Faculty of Medicine, University of New South Wales, Sydney, New South Wales, Australia (D.F.)

**Keywords:** cardiovascular disease, genetic variants, nonsynonymous, prediction

## Introduction

These are exciting times, with a plethora of new technologies that are expediting discovery of the genetic underpinnings of human disease. Comprehensive resequencing of the human genome is now feasible and affordable, allowing each person's entire genetic makeup to be revealed. The major focus of attention in genetics studies has been the small portion (1%) of the human genome that comprises the protein-coding sequences in genes (the “exome”), and the majority of causal disease-associated variants identified to date have been located in these regions.^1^ A remarkable extent of genetic variation in the protein-coding regions has been found, with at least 20 000 single-nucleotide polymorphisms (SNPs) present even in normal healthy subjects.^2,3^ Half these SNPs are nonsynonymous changes that result in an amino acid substitution that could potentially affect protein function. The greatest challenge now facing investigators is data interpretation and the development of strategies to identify the minority of gene-coding variants that actually cause or confer susceptibility to disease. To address this problem, bioinformatics tools have been developed to predict the likelihood of pathogenicity. A bewildering array of options is available, and users need to be aware of the programs most suited to their needs as well as the strengths and weaknesses of the various methods employed.

Here, we provide an introductory overview of some commonly used pathogenicity prediction programs as well as a set of illustrative cardiac examples. This article is tailored for readers who are not bioinformatics experts and is relevant to cardiovascular researchers undertaking human genetics studies as well as to clinicians performing genetic testing. For comprehensive reviews of available methods,^4–8^ detailed technical explanations of the bioinformatics and validation of individual programs,^9–21^ and comparative analyses in large variant data sets,^22–28^ we refer the reader to excellent articles published elsewhere. The important “take-home” message is that although bioinformatics prediction programs are extremely useful, the results cannot necessarily be taken at face value because all programs have inherent limitations, and additional supporting evidence is required to confirm that predicted deleterious variants have a role in disease processes.

## Importance of Gene Coding Sequence Variants in Human Disease

The Human Gene Mutation Database (HGMD)^1^ currently lists more than 120 000 variants in more than 4400 genes that have been associated with human diseases. Disease-associated variants include nonsense variants (amino acid changes that result in a stop codon), variants that create or abolish splice donor or acceptor sites, and insertions or deletions (indels) that shift the protein reading frame. All these types of variants have a high probability of altering protein function. Interpretation of missense SNPs (that change an amino acid but do not result in a stop codon) is far less straightforward and more difficult to predict because of the range of effects they can impart. Missense SNPs in critical residues can have disastrous consequences on protein function or structure. However, missense SNPs may be benign when the amino acid is substituted for another with similar biochemical properties, if the substitution occurs in an evolutionarily nonconserved position, or when the residue is not in a critical structural or functional domain of the protein. The average white individual has ≍10 000 missense SNPs in their exome, of which ≍200 are novel.^3^ Experimentally elucidating the consequences of each variant using in vitro studies and animal models is the best way to demonstrate functional effects, but this is impractical on a large scale. Reliable and high-throughput methods for evaluating missense SNPs are clearly required.

## Steps in Sequence Analysis

A number of different strategies may be used in genetics studies, and the choice of method depends on the population under investigation and the specific questions being addressed. Studies of Mendelian traits in large family kindreds have traditionally involved linkage analysis to define a chromosomal disease locus, followed by resequencing of candidate genes that are located within the interval. In cohorts of small families in which linkage is unable to be done, resequencing of selected candidate genes is often performed. These approaches have led to the discovery of numerous disease genes for a wide range of cardiac (and extracardiac) disorders and have provided a basis for commercial genetic testing (discussed in a later section). Whole-genome and whole-exome massive parallel sequencing platforms are now rapidly gaining popularity for discovery of new disease genes and for identification of variants in known disease genes in families. In cohorts of unrelated patients, resequencing of single genes and genome-wide association studies with SNP arrays have been used to look for rare and common variants that affect disease risk. Although cost is still a factor in large cohort studies, next-generation sequencing will undoubtedly be used increasingly in this setting.

**Figure 1.**
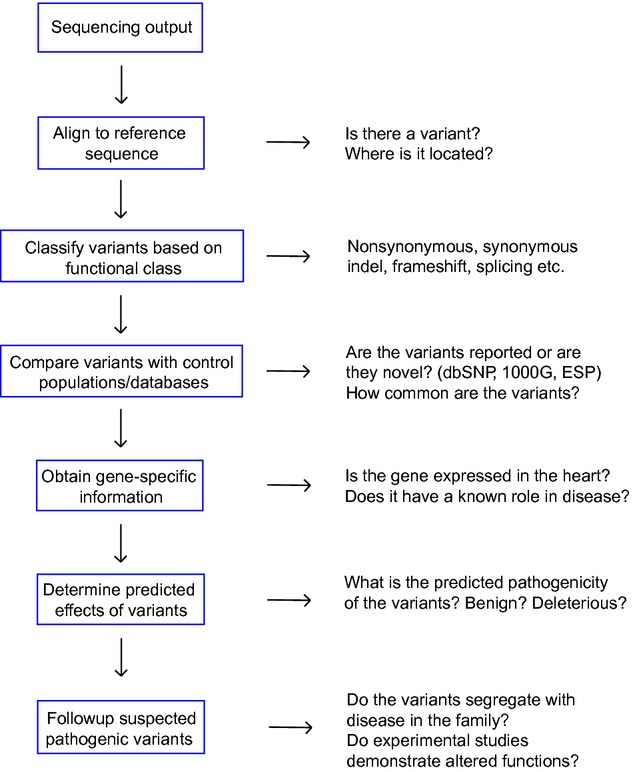
Flow chart showing steps for DNA sequence analysis. ESP indicates Exome Sequencing Project; 1000G, 1000 Genomes project.

Irrespective of the sequencing method used, the principles of sequence analysis are essentially the same (Figure 1). First, the sequencing output needs to be aligned to a human reference assembly to determine whether there are any differences with the “normal” sequence and to determine the location of variations (gene exon, gene intron, intergenic). Second, the potential effects of variants on the encoded protein need to be determined (eg, nonsynonymous or synonymous amino acid substitution, splice variant, indel, etc). Third, a search is made of publicly available databases, such as dbSNP, 1000 Genomes, and the Exome Sequencing Project, and in some cases, a cohort of healthy control DNA samples may be genotyped to determine whether variants are novel or have been previously reported and the prevalence of the variant allele. Some inferences then need to be made about potential functional effects. For cardiovascular diseases, variants in genes that are expressed in the heart or vasculature and that have relevant functions for the trait under study can be prioritized. However, it is important not to disregard the possibility that cardiac expression or function of some genes may not be recognized. Even after these filtering methods are employed, a long list of “suspicious” variants is likely to remain, and prediction tools have a key role in short-listing these for further analysis. Bioinformatics tools are heuristic, that is, they combine various types of parameters from multiple sources to infer likely pathogenicity when detailed experimental evaluation of individual variants is unavailable.

## Prediction Methods Available

In this review, we have looked at 8 of the currently available prediction tools for nonsynonymous variants to highlight aspects of how these types of programs work and their relative performance. The methods used and parameters assessed in these 8 programs are summarized in Table 1, with some useful notes about inputs and outputs in Table 2.

**Table 1.** Characteristics of 8 Commonly Used Gene Variant Functional Prediction Programs

Programs	Web Site	Method	Parameters Used	Training Data	Reference
PANTHER	http://www.pantherdb.org/	Hidden Markov Model	Evolutionary conservation across multiple protein families	Disease-associated mutations from HGMD; presumed neutral variants in dbSNP	^9,10^

SIFT	http://sift.jcvi.org/	Conservation of protein homologues	Evolutionary conservation	1 Retroviral+2 bacterial mutagenesis data sets; 5218 human disease-associated SNPs in Swiss-Prot; 3084 SNPs in dbSNP	^11–13^

Align-GVGD	http://agvgd.iarc.fr/	GV, GD	Evolutionary conservation+biochemical properties (amino acid composition, polarity, volume)	Concurrence of unclassified variants with deleterious mutations in *BRCA1*; 1514 nonsynonymous SNPs in *TP53* gene	^14,15^

PMut	http://mmb2.pcb.ub.es:8080/PMut/	Neural network	Evolutionary conservation+structural effects (secondary structure and solvent accessibility)	9334 human disease-associated mutations in 811 proteins from Swiss-Prot; 11 372 neutral variants from *Escherichia coli* mutagenesis data set+811 mutation-associated proteins	^16^

SNPs3D	http://www.snps3d.org/	Support vector machine	Evolutionary conservation+structural effects (protein folding)	Monogenic disease data from HGMD; 10 263 disease SNPs in 731 genes; 16 682 control SNPs	^17,18^

PolyPhen-2	http://genetics.bwh.harvard.edu/pph2/	Naive Bayes classifier	Evolutionary conservation+structural effects*	2 Training models: Hum Div (3155 Mendelian disease-causing variants in UniProt; 6321 presumed nondamaging SNPs) and Hum Var (13 032 human disease-causing mutations from UniProt; 8946 common human nsSNPs with no link to disease)	^19^

MutPred	http://mutpred.mutdb.org	Random forest	Evolutionary conservation†+structural effects‡+predicted functions	26 655 Disease-associated mutations in HGMD; 23 426 presumed neutral SNPs in Swiss-Prot	^20^

SNPs&GO	http://snps-and-go.biocomp.unibo.it/snps-and-go/	Support vector machine	Evolutionary conservation+local sequence+gene ontology score	16 330 Disease-associated SNPs from Swiss-Prot; 17 432 presumed neutral SNPs from Swiss-Prot	^21^

GD indicates Grantham deviation; GV, Grantham variation; HGMD, human gene mutation database^1^; MSA, multiple sequence alignment; SNP, single-nucleotide polymorphism.

* PolyPhen2 uses 8 sequence-based and 3 structure-based features, including position-specific independent count score of wild-type allele, differences in this score between the wild-type and variant alleles, number of residues observed at the position in the MSA, residue side-chain volume change, variant position with respect to a protein domain defined by Pfam, variant allele congruency to MSA, sequence identity with closest homologue deviating from wild-type allele, normalized accessible surface area of amino acid residue, crystallographic β-factor, and change in accessible surface area propensity for buried residues.

† SIFT score, Pfam profile score, and transition frequency (likelihood of observing a given SNP in the UniRef80 database and Protein Data Bank).

‡ Predicted secondary structure, solvent accessibility, transmembrane helices, coiled-coil structure, stability, B-factor, and intrinsic disorder.

**Table 2.** Input and Output Characteristics for 8 Common Prediction Algorithms

Programs	Input	Access to Intermediate Information	Output	Program-Recommended Pathogenicity Criteria
PANTHER	WT protein sequence (FASTA or plain format), variant/s of interest; MSA is program generated	MSA (and phylogenetic tree)	subPSEC score: 0 (benign) to −10 (most deleterious); *P*_del_: 0 (0%) to 1.0 (100%)	subPSEC score: <−3 (50% likelihood of deleterious effects); *P*_del_ >0.5

SIFT	WT protein sequence (FASTA format) or Clustal-formatted MSA (WT query sequence must appear first in MSA), variant/s of interest; MSA is program- or user generated	MSA (if single query sequence inputted)	Scaled probability score: 0 (most deleterious) to 1 (benign); no. sequences at position; median sequence conservation	Scaled probability score: <0.05

Align-GVGD	FASTA-formatted MSA* (WT query sequence must appear first in MSA), variant/s of interest	No	Combined GV+GD risk estimate: C0 (lowest risk) to C65 (highest risk); individual GV and GD scores	Incremental risk estimates: 1.0- (C0) to >4.0-fold (C65)

PMut	WT protein sequence (FASTA or plain format), or FASTA-formatted MSA (WT query sequence must appear first in MSA), variant/s of interest	PSI-BLAST raw output (protein family analysis), MSA (FASTA format), PHD raw output (secondary structure and accessibility predictions)	Qualitative prediction: neutral or pathogenic; pathogenicity index: 0 (low) to 1.0 (high); reliability: 0 (low) to 9 (high)	Pathogenicity index: >0.5; reliability: >5

SNPs3D	dbSNP, RefSNP or sequence accession number (if variant not present in results list, select protein accession and enter mutation manually); MSA is program generated	MSA	SVM score: positive (nondeleterious) or negative (deleterious)	Negative SVM score

PolyPhen-2	WT protein sequence (FASTA format) or protein identifier, variant position, WT and variant amino acids; MSA is program generated unless downloaded stand-alone version used to input user-generated MSA	MSA, 3D visualization (if protein structure information available)	Qualitative prediction: benign, possibly damaging, probably damaging; Hum Div/Hum Var scores: 0 (benign) to 1.0 (most deleterious); sensitivity: 0 (low) to 1.0 (high); specificity: 0 (low) to 1.0 (high)	Probably damaging prediction; HD/HV scores: closer to 1

MutPred	WT protein sequence in FASTA format, variant/s of interest; MSA is program generated	No	“*g*” score: 0 (low) to 1 (high); “*p*” score: 0 (low) to 1 (high)	Possibly deleterious (*g*>0.5), probably deleterious (*g*>0.75)

SNPs&GO	UNIPROT accession number, variant position, WT and variant amino acids; MSA is program generated	No	Qualitative prediction: neutral or disease related; reliability index: 0 (unreliable) to 10 (reliable)	Disease prediction; reliability index: >5

General (“*g*”) score indicates probability that an amino acid substitution is deleterious; MSA, multiple sequence alignment; property (“*p*”) score, statistical likelihood (*P* value) that structural and functional properties will be altered; P_del_, deleterious probability; PHD, Profile fed neural network systems from Heidelberg; PSI-BLAST, Position-Specific Iterated Basic Local Alignment Search Tool; subPSEC, substitution position-specific evolutionary conservation score, estimated from the negative logarithm of the probability ratio of wild-type and mutant amino acids at a specific position; WT, wild type.

* Except for 7 tumor-related genes in program library.

Genome sequences that are highly conserved during evolution are thought to be important for protein function, and disease-associated mutations tend to be abundant at these sites.^4,5^ Many programs, including PANTHER (Protein Analysis Through Evolutionary Relationships)^9,10^ and SIFT (Sorts Intolerant From Tolerant amino acid substitutions),^11,12,13^ rely primarily on the extent of sequence conservation of a specific residue, which is assessed by looking at an alignment of the sequences of this region of the protein across a wide range of different species, that is, multiple sequences alignment (MSA). Many programs take factors in addition to evolutionary conservation into consideration. Align-GVGD^14,15^ also looks at the effects of differences that an amino acid substitution would have on the biochemical properties of a residue, such as changes in volume, polarity, and charge. The Grantham Variation (GV) score component of Align-GVGD reflects the extent of biochemical variation among amino acids at a given position within an MSA, whereas the Grantham Deviation (GD) score reflects the biochemical distance between variant and wild-type amino acids at a given residue. Several programs, including PMut,^16^ SNPs3D,^17,18^ and PolyPhen-2,^19^ use varying combinations of sequence-based and protein structure-based features, such as the effect of a variant on protein folding and accessible surface area of the amino acid residue. MutPred^20^ is an extension of SIFT that differs most significantly from other programs by its incorporation of predicted functional sites, including DNA-binding residues, catalytic residues, calmodulin-binding targets, and predicted posttranslational modification (phosphorylation, methylation, ubiquitination, glycosylation) sites. A broad range of additional parameters are also included in SNPs&GO,^21^ with evaluation of evolutionary data from PANTHER, the sequence environment of a residue (including 18 residues on either side of the variant residue), and a gene ontology (GO) score that derives information about the biological processes, cellular components, and molecular functions of gene products in different species from the GO database. These prediction tools have been benchmarked on large mutation data sets, and although developed for use in classifying human mutations, some of these programs can be applied to bacteria, plants, and other organisms.^29^

## Example Variants

To further illustrate some of the features of these programs, we used them to make predictions about 18 missense variants that we selected as examples, including 9 rare variants that have robust genetic or functional evidence to implicate them as disease causing in various cardiomyopathies and arrhythmias,^30–37^ and 9 common variants implicated in disease susceptibility (Table 3).^38–46^ The results of these predictions are shown in Table 4. For the 9 rare variants, the number of variants that were accurately predicted as likely to be deleterious ranged from 2 using PANTHER (22%, although predictions were able to be made for only 4 variants) to 8 (89%) with SIFT, PolyPhen-2, MutPred, and SNPs&GO. The greatest variability was seen with 2 programs, PANTHER and Align-GVGD, and 3 variants, R403Q *MYH7*, R92Q *TNNT2*, and D175N *TPMI*. For the 9 common variants, with a few exceptions, predictions were overwhelmingly neutral. A closer examination of the factors on which the predictions are based helps to explain these results.

**Table 3.** Nonsynonymous Variants Associated With Cardiac Disorders

Gene	Protein	Variant	Location	Clinical Association	Genetic Evidence	Functional Evidence	Reference
Rare variants							

*LMNA*	Lamin A/C	N195K	Coiled-coil rod domain	DCM	Family	Yes	^30^

*MYH7*	β-Myosin heavy chain	R403Q	Myosin head, interacts with actin	HCM	Family	Yes	^31^

*MYH7*	β-Myosin heavy chain	S532P	Actin-binding domain	DCM	Family	Yes	^32^

*TNNT2*	Cardiac troponin T	R92Q	α-Tropomyosin-binding domain	HCM	Family	Yes	^33^

*TNNT2*	Cardiac troponin T	R141W	α-Tropomyosin-binding domain	DCM	Family	Yes	^34^

*TPMI*	α-Tropomyosin	D175N	Troponin T–binding domain	HCM	Family	Yes	^35^

*KCNQ1*	KCNQ1	S140G	S1 transmembrane domain	AF	Family	Yes	^36^

*KCNQ1*	KCNQ1	Y315S	Pore-forming domain	LQTS	Family	Yes	^37^

*KCNH2*	HERG	G628S	Pore-forming domain	LQTS	Sporadic	Yes	^37^

Common variants							

*MYH6*	α-Myosin heavy chain	A1101V	Coiled-coil rod domain	HR, PR	Case–control	No	^38^

*AGT*	Angiotensinogen	M235T	Polypeptide chain	HT	Case–control	Yes	^39^

*NOS3*	Endothelial NO synthase	E298D	NOSIP interaction region	AF, CAD	Case–control	Yes	^40^

*KCNH2*	HERG	K897T	Intracellular C-terminal domain	LQTS, AF	Case–control	Yes	^41,42^

*KCNE1*	KCNE1	S38G	Extracellular N-terminal domain	AF	Case–control	Yes	^43^

*SCN5A*	Cardiac sodium channel	H558R	Intracellular repeat I/II linker	AF	Case–control	Yes	^44^

*ADRB1*	β1-adrenergic receptor	S49G	Extracellular N-terminal domain	HR, DCM	Case–control	Yes	^45^

*ADRB1*	β1-adrenergic receptor	G389R	Intracellular C-terminal domain	HF, AF	Case–control	Yes	^45^

*CYP2C9*	Cytochrome P450 2C9	I359L	Substrate recognition site 5	Warfarin dose	Case–control	Yes	^46^

AF indicates atrial fibrillation; CAD, coronary artery disease; DCM, dilated cardiomyopathy; HCM, hypertrophic cardiomyopathy; HF, heart failure; HR, heart rate; HT, hypertension; LQTS, long QT syndrome; NO, nitric oxide; NOSIP, eNOS interacting protein; PR, PR interval.

**Table 4.** Predicted Effects* of Rare and Common Nonsynonymous Variants

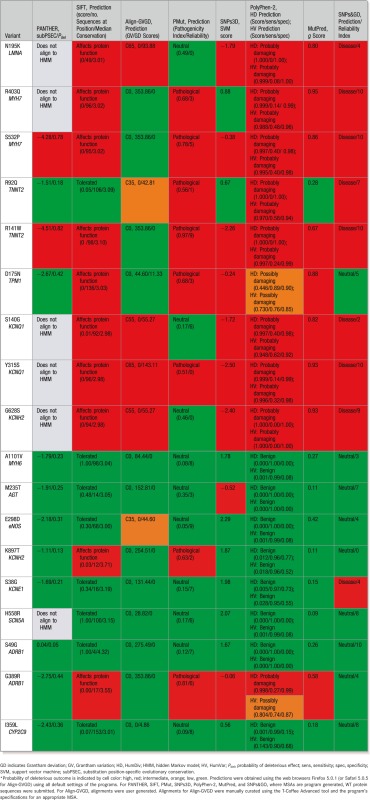

## Key Role of Amino Acid Conservation in Predicting Pathogenicity

As noted above, sequences that are highly conserved across species are often functionally important, and high prediction success has been achieved for algorithms that predominantly use evolutionary-based information.^9–13^ Sequence-based methods do have their limitations,^47^ and this is demonstrated by the predictions generated by PANTHER and Align-GVGD. Although PANTHER is generally reliable when predictions are obtained,^26^ it failed to generate predictions for 6 of the 18 variants in our example data set. This may occur if the sequence alignment is poor or when a variant is located at a residue that is not present in a majority of species and hence is unable to be modeled in a Human Markov Model. In Align-GVGD, we found wide discordance between sequence conservation (GV) and biochemical change (GD) components for several variants that resulted in a neutral prediction. Sequence conservation appeared to have relatively less weighting than biochemical change because neutral predictions were more likely to be obtained when the GV scores were high and the GD scores were zero (eg, R403Q *MYH7*, S532P *MYH7*), rather than the converse situation with low GV and high GD scores (eg, N195K *LMNA*, Y315S *KCNQ1*). As a general concept, adding protein structural or functional parameters should provide greater predictive accuracy than consideration of sequence conservation alone,^27^ but this only applies when protein structure or function is known and the relevant databases are up to date. Quite commonly, this information is incomplete or lacking, and the predictions have to rely predominantly on the evolutionary conservation component.

## The Importance of MSAs in Predictions

The number of species in an MSA and the evolutionary distance between them heavily influence algorithm accuracy. Evolutionary depth in MSAs is recommended because this potentially provides more information about the extent of conservation. If sequences in the MSA are too similar (eg, dog, pig, human), then variants not normally imparting a functional consequence on the protein will tend to be classified as pathogenic. On the other hand, comparing a broader range of species, such as small rodents (rat, mouse), zebra fish, fly, and worm, may strengthen the case for a variant in a highly conserved residue being pathogenic, but may also produce false negatives if there is divergence in the protein sequences and biological functions of more distantly related species.^7^ Similarly, there are no clear indications about whether inclusion of different protein isoforms and different members of the same protein family will strengthen or weaken predictions. In 1 comparative study, PolyPhen-2 appeared to be least susceptible to differences in the MSAs, whereas Align-GVGD was highly susceptible and had a propensity to call variants as neutral when large numbers of sequences were utilized.^27^ It has been noted that programs do not always perform best with their own program-generated MSA and can have more accurate results with gene-specific MSAs that have been optimized by the user.

PANTHER, SNPs3D, MutPred, and SNPs&GO generate MSAs internally and do not allow the option of users creating and submitting their own MSAs. SIFT and PMut internally generate an alignment but also permit user-generated alignments. The Web-server version of Polyphen-2 has its own alignment pipeline, but user-generated alignments can be submitted to the stand-alone software version, which can be downloaded onto a local computer. Align-GVGD has a very limited set of alignments, so users mostly need to supply their own. This enables greater control of user-defined sequences in the alignment and flexibility of adding or removing sequences in the MSA, but entails considerable additional work to obtain and align the relevant protein sequences. There is also the real possibility of skewing the results by variations in the numbers and types of species selected to be included in the MSA.

MSAs can be obtained from the Pfam (protein families) database^48^ or manually curated and then aligned using freely available online alignment tools such as the more widely used programs ClustalW2,^49^ MAFFT,^50^ MUSCLE,^51^ PROMALS,^52^ and T-Coffee.^53^ Alignments produced by the different programs for specific regions can differ, however, and it has been suggested that more than 1 MSA program may be required, particularly for sequences that contain deletions or insertions. A number of scoring systems have been devised to assess the quality of MSAs, with the overall conclusion that, like the protein prediction programs available, a single flawless method is not available.^54–56^

## Location, Location, Location

Significant discrepancies between bioinformatics predictions and experimentally validated effects often arise because the functional characteristics of the region in which a variant is located are inadequately taken into account. Amino acid changes that have modest pathogenicity predictions may nevertheless have a substantial impact if they occur in critical regions of a protein, such as those involved in protein–protein interactions or posttranslational modification. Conversely, variants predicted to be pathogenic because of extensive biophysical modification of a residue may have no effects if this occurs in a relatively unimportant region. Although these issues are addressed in part by MutPred and SNPs&GO, which incorporate some functional parameters, lack of consideration of gene-specific functional effects is a universal limitation.

Examples of the importance of the protein “neighborhood” are provided by the R403Q *MYH7*, R92Q *TNNT2*, and D175N *TPMI* variants. The Arg403Gln mutation in the gene encoding myosin heavy chain (*MYH7*) causes hypertrophic cardiomyopathy in humans and in mice.^31^ The R403 residue is located in the myosin head adjacent to the actin-binding site and is invariant in myosin heavy chains in the heart and other tissues across a range of species from human to amoeba.^31^ Although this high degree of sequence conservation and the biophysical effects of loss of an arginine are able to be assessed in the prediction algorithms, none of the programs would have considered the key role of the 403 residue in actin–myosin interaction, calcium sensitivity, and energy utilization. A similar argument can be made for the R92Q *TNNT2* variant, which is in the elongated tail domain of cardiac troponin T at the site where the tropomyosin monomers overlap. This variant has been shown to have distinct effects on calcium sensitivity and thin filament sliding speed in vitro and results in a hypertrophic cardiomyopathy phenotype in mice,^32^ yet only 4 of the 8 programs used predicted it to be probably (n=3) or possibly (n=1) deleterious. The D175N *TPMI* variant, located in the troponin T–binding site in tropomyosin, was also only identified by 5 of the 8 programs as probably (n=4) or possibly (n=1) deleterious despite robust genetic and in vivo functional evidence of pathogenicity.^35^

## Rare Versus Common Variants

Genetic variation is being recognized increasingly to play a role in many cardiovascular disorders.^57,58^ At one end of the spectrum, single-gene variants that have a large functional effect have been considered sufficient to cause disease in families with Mendelian patterns of inheritance. These variants are typically rarely present in the general population, and many are “private” mutations seen only in 1 family. Single rare variants have been associated with numerous heritable cardiomyopathies and arrhythmias, including familial hypertrophic cardiomyopathy, familial dilated cardiomyopathy, arrhythmogenic right ventricular cardiomyopathy and long QT syndrome. In contrast, commonly occurring genetic variants have been associated with complex traits such as hypertension, coronary artery disease, diabetes, and atrial fibrillation (the common disease, common variant hypothesis). Common SNPs can be identified by genome-wide association studies in large cohorts of affected and unaffected individuals. These types of variants are potentially important because of their relatively high-population frequencies, although the risks associated with each variant may only be modest. Recently, human genome sequencing studies have heightened interest in the potential role of rare variants in common diseases.^3,59–63^ A new paradigm has been proposed in which the cumulative burden of unique personal combinations of rare variants may contribute substantially to the heritable component of complex disease.

These perspectives on the role of genetics need to be kept in mind when considering the performance of gene variant functional predictions. A striking finding in our example variants was the differences between predictions for rare and common variants. Whereas the known functional rare variants were correctly predicted by a majority of programs as deleterious, the common variants were mostly predicted as nondeleterious. There are several factors that might explain this discrepancy. First, it is important to note that common SNPs that show significant associations with disease in genomewide association studies are almost always not the causal variants themselves but are markers for a pathogenic SNP that is coinherited in the same haplotype. For example, A1101V *MYH6* was significantly associated with heart rate, and to a lesser extent with PR interval, in a study of more than 20 000 individuals.^38^ The uniformly neutral predictions for A1101V *MYH6* may in fact be correct if the trait is not directly attributable to this SNP. Patients carrying the M235T *AGT* SNP have increased plasma angiotensinogen levels and increased risk of hypertension.^39^ Although 1 program, SNPs3D, had a pathogenic prediction, the same argument can be made that M235T *AGT* might only be a marker of a risk allele. In contrast to the A1101V *MYH6* and M235T *AGT* SNPs, several of the variants in genes encoding cardiac ion channels have had direct experimental validation of deleterious effects. For example, K897T *KCNH2* changes the biophysical properties of the *I*_Kr_ current and also creates a new phosphorylation site for Akt protein kinase that inhibits channel activity.^41,42^ Despite these findings, only 2 of the 8 programs (SIFT, PMut) predicted pathogenic effects. Even MutPred, which includes posttranslational modification site prediction, did not call this SNP as pathogenic. S38G *KCNE1* has loss-of-function effects on *I*_Ks_,^43^ whereas H558R *SCN5A* is a potent modifier of *I*_Na,_ with effects that vary with different genetic backgrounds.^44^ SNPs&GO predicted S38G *KCNE1* as pathogenic, but all other programs predicted both variants to be neutral. These differences between predictions and experimental data for ion channel variants may be a result of the locations of these variants in gene-specific functional domains that are not taken into consideration by prediction algorithms (as noted above). Alternatively, these findings may indicate that bioinformatics tools are relatively better at predicting pathogenic rare variants that have large functional effects than common variants that have more modest functional effects.

## Which Method Is Best?

Most of the prediction programs have been benchmarked by their curators using large variant data sets and have been shown to perform well (Table 1). However, there are relatively few studies that have systematically compared the predictive accuracy of different programs in the same test data set. This can be a difficult exercise because the various types of outputs may not be readily standardized. In addition, because each of the programs obtains sequence and/or structural information from different databases, there may be confounding factors of conflicting or missing information. Also, if a data set for testing a program's accuracy is similar to its training data set, bias occurs_,_ and misleading inferences of a program's superior performance can arise. The creators of PMut even state that its algorithm was trained using alignments in the Pfam Database, so better prediction performance is expected toward Pfam alignments.^16^

The results of 5 comparative studies are shown in Table 5. Chan and colleagues^22^ evaluated 254 missense variants using SIFT, PolyPhen, Align-GVGD, and the BLOSUM62 matrix. The overall accuracies (algorithm based on the sum of true-positive and true-negative rates) for single programs were not dissimilar, ranging from 73% (Align-GVGD) to 82% (SIFT). It was noted that the programs with higher sensitivity detected more deleterious variants but had lower specificity, whereas programs with lower sensitivity but high specificity better predicted neutral variants and had fewer false positives for deleterious variants. Wei and colleagues^24^ looked at 204 variants with 6 programs and concluded that SIFT and PolyPhen were the overall top predictors, followed by nsSNPAnalyzer. Hicks and colleagues^27^ found that SIFT, Align-GVGD, PolyPhen-2, and Xvar had similar overall accuracy when optimal MSAs were provided for each program. Align-GVGD had a very low median sensitivity (10%) and high median specificity (>95%), but these results were considered unreliable, given the bias for negative predictions with large MSAs. Because Align-GVGD performed best with a manually curated MSA, it was considered less suitable for use in large-scale sequencing analyses. The speed of the program and the number of variants that can be inputted are other criteria that limit the suitability of most programs for use in next-generation sequencing analysis. To meet these needs, Schwarz and colleagues have developed MutationTaster.^25^ When compared with PANTHER, PolyPhen, Poly Phen-2, PMut, and SNAP, in a training set of 1000 disease-linked variants and 1000 SNPs, MutationTaster was found to have the highest accuracy (86%) and was substantially faster than the other programs studied. In the most comprehensive analysis to date, Thusberg and colleagues^26^ utilized 9 programs to evaluate more than 40 000 variants in several databases, including dbSNP. PhenCode, LSDBs (locus-specific mutation databases), and IDbases (LSDBs for immunodeficiency-causing mutations). These authors concluded that no single method could be rated as best by all parameters but that SNPs&GO and MutPred were overall superior to other programs tested.

**Table 5.** Studies Comparing Performance of Different Prediction Algorithms

Programs Tested	Variants Evaluated	Sensitivities (True-Positive Rates)	Specificities (True-Negative Rates)	Overall Accuracy*	Reference
SIFT, PolyPhen, Align-GVGD, BLOSUM62	254 Missense variants in 5 genes involved in familial cancer syndromes and noncancer genetic disease	SIFT (84%), Polyphen (83%), BLOSUM62 (75%), Align-GVGD (69%)	BLOSUM62 (85%), Align-GVGD (84%), SIFT (77%), PolyPhen (58%)	SIFT (82%), BLOSUM62 (78%), PolyPhen (76%), Align-GVGD (73%)	^22^

SIFT, PolyPhen, PMut, SNPs3D, PhD-SNP, nsSNPAnalyzer	204 Variants in the human cystathionine β synthase gene	SIFT (89%), PolyPhen (87%; if “possibly damaging” variants were grouped as deleterious), SNPs3D (82%), nsSNPAnalyzer (80%), PhD-SNP (70%), PMut (44%)	PMut (79%), PolyPhen (70%; if “possibly damaging” variants grouped as neutral), nsSNPAnalyzer (59%), PhD-SNP (53%), SIFT (52%), SNPs3D (47%)	PolyPhen (71%; if “possibly damaging” variants grouped as neutral), nsSNPAnalyzer (67%), PMut and SIFT (66%), SNPs3D (61%), PhD-SNP (59%)	^24^

SIFT, Align-GVGD, PolyPhen-2, XVAR	267 Variants in 4 cancer-susceptibility genes	Median sensitivities: Xvar (98%), PolyPhen-2 (90%), SIFT (85%), Align-GVGD (10%)	Median specificities: Align-GVGD (>95%), SIFT (52%), PolyPhen-2 (40%), Xvar (33%; if *TP53* gene excluded)	Align-GVGD, PolyPhen-2, and Xvar (79%), SIFT (77%)	^27^

MutationTaster, PolyPhen, PolyPhen-2, SNAP, PANTHER, PMut	1000 Disease-associated mutations and 1000 polymorphisms	MutationTaster (86%), PolyPhen and PolyPhen-2 (78%), SNAP (69%) PMut (68%), PANTHER (50%)	MutationTaster (86%), PolyPhen-2 (83%), PolyPhen (74%), SNAP (69%), PMut (63%), PANTHER (52%)	MutationTaster (86%), PolyPhen (76%), PolyPhen-2 (72%), PMut (65%), SNAP (60%), PANTHER (35%)	^25^

MutPred, nsSNPAnalyzer, PANTHER, PhD-SNP, PolyPhen, PolyPhen-2, SIFT, SNAP, SNPs&GO	More than 40 000 variants from dbSNP, PhenCode, LSDBs, IDbases	SNAP (88%), PolyPhen-2 (86%), MutPred (85%), PANTHER (77%), PolyPhen (74%), SNPs&GO (71%), SIFT (68%), PhD-SNP (63%), nsSNPAnalyzer (61%)	SNPs&GO (92%), PolyPhen (85%), PhD-SNP (79%), MutPred (78%), PANTHER (76%), PolyPhen-2 (70%), SIFT (62%), nsSNPAnalyzer (58%), SNAP (56%)	SNPs&GO (82%), MutPred (81%), PANTHER (76%), SNAP (72%), PhD-SNP and PolyPhen-2 (71%), PolyPhen (70%), SIFT (65%), nsSNPAnalyzer (60%)	^26^

IDbases indicates LSDBs for immunodeficiency-causing mutations; LSDB, locus-specific databases.

* Estimate of true positives and true negatives, some variations in formulas used in different publications.

## Consensus Predictions

Several groups have proposed that using the consensus predictions of a number of programs may be more reliable than using a single program.^22–24^ For example, in the analysis by Chan and colleagues,^22^ the 4 programs tested gave concordant results for only 63% of the variants. However, when this occurred, the overall predictive value increased to 88%. Similarly, Wei and colleagues^24^ found that when different combinations of programs were used, the consensus of 5 programs (SNPs3D excluded) gave the best total accuracy (73%). In our example variants, we found that no program predicted all rare variants as pathogenic. Seven of the 9 rare variants had consensus predictions by SIFT and PolyPhen-2_,_ and all 9 rare variants were identified correctly as deleterious when other combinations of 2 methods were used, for example, SIFT and PolyPhen-2 or MutPred or SNPs&GO. For the 9 common variants, with the exception of G389R *ADRB1*, the combined predictions of multiple programs did not increase the number of positive predictions.

Although confidence in a result may be increased if concordant results are obtained with a number of programs, some pathogenic variants may be missed. On the other hand, having less stringent criteria, such as requiring any 1 program to be deleterious, will increase the chances that all the true positives will be detected but may also result in more false-positive results. A further consideration is that output similarities may be consequences of the similarity of inputs for some combinations of programs and do not necessarily equate with greater prediction accuracy.

The comparative studies outlined above have been benchmarked using variants that have been predetermined to be deleterious or benign. The performance of these methods on a genomewide scale in which there are many thousands of variants of unknown function has been less extensively evaluated. Chun and Fay compared SIFT and PolyPhen with their likelihood ratio test (LRT) in an evaluation of 3 human genomes.^23^ Surprisingly, 76% of variants were predicted as deleterious by only 1 program_,_ and only 5% of variants were predicted as deleterious by all 3 programs. These authors proposed that it was the small proportion of variants with consensus predictions that was most likely to be functionally significant. This is a very important point that warrants further validation. Although using multiple prediction programs for each variant is desirable, this is time consuming and impractical on a large scale. To address this issue, Liu and colleagues have recently developed dbNSFP (database for nonsynonymous SNPs' functional prediction).^28^ This method integrates pathogenicity predictions from SIFT, PolyPhen-2, LRT, and MutationTaster into a single application.

## Recommendations

The selection of pathogenicity prediction programs depends very much on the situation and the type of data being interrogated. When there are only a small number of specific variants under consideration, for example, in a family that has undergone linkage analysis and sequencing of candidate genes in a disease interval or with a family in which genetic testing of known disease genes has been performed, a detailed analysis is warranted_,_ and it is highly recommended that a number of prediction programs be used. We have routinely used SIFT, PolyPhen-2, PMut, and SNPs&GO and have recently added MutationTaster to our suite of preferred programs. The selection of programs is probably less critical than looking at consensus predictions (when all programs agree) or majority predictions (when most programs agree). At present, only a subset of programs (including SIFT, PolyPhen-2, and MutationTaster) have batch modes that allow multiple variants to be simultaneously inputted and are suitable for analyzing large next-generation sequencing data sets. In the next few years, it is likely that many more programs will be adapted for this use.

## Use of Gene Variant Prediction Programs in Genetic Testing

Genetics studies in families have generally been performed by research groups seeking to decipher molecular mechanisms of disease. As a result of these studies, lists of disease genes have been established for many of the inherited cardiomyopathies and arrhythmias. Commercial genetic testing of subsets of the more common of these disease genes is now available_,_ and expert consensus recommendations for indications for genetic testing have recently been compiled by the Heart Failure Society of America, the Heart Rhythm Society, and the European Heart Rhythm Association.^64,65^ Healthcare professionals are now empowered to send off patient DNA samples for genetic testing_,_ and informed interpretation of the results is crucial.

If the results for a family proband DNA sample come back as positive, showing a variant in gene X that is “probably pathogenic,” it cannot necessarily be assumed that this is *the* disease-causing mutation in the family_,_ and a number of questions need to be asked initially along the lines of the flowchart in Figure 1. One needs to know whether the variant is novel, rare_,_ or commonly present in a population whose ethnicity is similar to that of the family being studied. As noted above, disease-causing mutations are nearly always rare and are often novel. The genes on genetic testing panels have all been preselected on the basis of known associations with cardiac disease, but it is useful to know whether the same variants, other variants at the same amino residue, or variants in neighboring residues in these genes have previously been identified with the same disorder or other cardiac disorders. This information can be obtained by searching mutation databases or the published literature. Bioinformatics tools have undoubtedly been used to come to the “probably pathogenic” annotation, and it is useful to know which programs and how many programs were used and the criteria used to define pathogenicity. We now know that every individual carries hundreds of novel potentially pathogenic variants,^3,66^ and so additional steps should be taken to make a case for a particular variant being disease causing. Determining whether a variant cosegregates with disease status in a family is a key factor in assessing its likely role in disease. Clinical evaluation of all first-degree relatives of an index case with suspected heritable disease should be performed and blood samples taken for DNA analysis. The presence or absence of a variant in family DNA samples can be readily ascertained by simple tests, such as polymerase chain reaction and sequencing. Factors such as variable expressivity and penetrance and phenotype phenocopies need to be taken into account when assessing variant segregation in a family. Even if a variant does cosegregate with the family phenotype, however, this cannot be regarded as definitive evidence of disease causation. The final interpretation of clinical significance relies on a considered balance of probabilities and is ideally performed in the setting of a multidisciplinary clinic in which pretest and posttest genetic counseling is provided. The role of genetics in clinical practice is likely to increase exponentially in the near future as whole-genome sequencing to document personal genomes becomes more readily available. This type of information will take genetics beyond looking for rare disease-causing variants in families to assessment of a single patient's risk of developing common diseases and responses to drug therapies.^67^

## Future Directions

This is a rapidly moving field_,_ and the need for faster and more comprehensive prediction tools is growing in parallel with the exponential use of next-generation sequencing. In the short term, submission inputs/outputs for prediction programs need to be streamlined, database resources need to be updated and maintained, quantitative and standardized measures of accuracy and reliability are required, and gene-specific functional domain information should be taken into account. In addition to refining methods to assess nonsynonymous variants, there is an ongoing need to look at other types of variants and parameters. VAAST, developed by Yandell and colleagues,^68^ has been recently developed specifically to analyze next-generation sequencing data and includes scoring of a broad range of coding and noncoding genetic variants, as well as incorporation of pedigree data. Comprehensive programs such as this will be invaluable for looking at the role of rare variants in both rare and common disorders. A generic limitation of all programs is the focus on single variants, and future refinements of genomic prediction tools would ideally incorporate evaluation of clusters of variants and their interactions.^8,69^ The extent to which the cardiac “environment” can affect gene variant effects is also an important question.^70^ The development of integrative strategies that can delineate unique individual cardiac substrates for disease is a daunting task but will ultimately be required to successfully implement personalized approaches to medical diagnosis and management.
